# Study on the mechanism of action of Kangfuxin liquid in alleviating DSS-induced ulcerative colitis in mice

**DOI:** 10.3389/fphar.2026.1823526

**Published:** 2026-05-28

**Authors:** Yanjun Yue, Na Sai, Xiaomei Qu, Xiao Qi, Zhibin Xiao, Ruiwen Shi, Ying Zhang, Jing He, Yue Zhang, Yongzai Qiang, Haibin Guan

**Affiliations:** 1 College of Pharmacy, Inner Mongolia Medical University, Hohhot, China; 2 Department of Pharmacy, Affiliated Hospital of Inner Mongolia Medical University, Hohhot, China

**Keywords:** 16S rRNA, gut microbiota, Kangfuxin, metabolomics, ulcerative colitis

## Abstract

In this study, we investigated the potential regulatory mechanisms of KFX on Dextran Sulfate Sodium Salt (DSS)-induced UC in mice by 16 S rRNA gene sequencing and metabolomics analysis of colonic contents. Differentially expressed metabolites and metabolic pathways were detected using a colon contents metabolomics-based approach. 16 S rRNA gene sequencing was used to assess changes in gut microbes at the genus level and for functional prediction. Results revealed metabolic disturbances in UC mice associated with 33 bacterial genera and 70 metabolites in the gut. Collectively, these findings suggest that UC is associated with metabolic disorders and microbial dysbiosis. Further, our data indicate that KFX administration is accompanied by significant alterations in intestinal flora composition and metabolic profiles, which may contribute to its protective effects against UC. However, the causal relationships between these microbial and metabolic changes and the therapeutic effects of KFX remain to be established.

## 1 Introduction

Ulcerative colitis (UC) is a chronic recurrent inflammatory disease of the intestinal tract (Clua-Ferré et al., 2024). The site of UC pathogenesis is primarily focused on the colonic mucosa and can diffusely damage the rectum. The typical clinical symptoms of UC are weight loss, fatigue, fever, diarrhoea, abdominal pain, and rectal bleeding (Goens and Micic, 2020). Patients with UC usually go through intermittent phases of deterioration and remission (Zallot and Peyrin-Biroulet, 2013). As the disease develops and progresses during the treatment period, patients are at higher risk of developing associated complications such as intestinal fibrosis (Wang et al., 2022), increased risk of developing colon cancer (Jess et al., 2012) and extraintestinal manifestations (Vavricka et al., 2015). The pathogenesis of UC has not yet been fully elucidated, but it is thought to be multifactorial and the result of complex interactions between genetic susceptibility, environmental factors, immune response and intestinal flora dysbiosis (Nishida et al., 2018). Initially, the incidence and prevalence of UC was significantly higher in Western countries than in the rest of the world, and although the incidence in Western countries has stabilised, the prevalence is still more than 0.3% (Ng et al., 2017), and with the spread of dietary cultures and Western lifestyles, the incidence and prevalence of UC in newly industrialised countries have shown an increasing trend year by year, and the global burden is facing a great challenge.

Clinical treatment of UC is mainly based on drug therapy, such as 5-aminosalicylic acid (mesalazine), adrenocorticotropic hormones (hydrocortisone), antibiotics (metronidazole) and immunosuppressive agents (Liang et al., 2024). However, these drugs do not completely cure the disease, have unstable efficacy, are prone to relapse, have many adverse effects and have multiple complications (Xu et al., 2004). During the acute phase of UC activity, proinflammatory cytokines further trigger the production of oxidation products, which disrupt the redox balance and exacerbate inflammation by inducing redox-sensitive factors, ultimately leading to the destruction of the intestinal barrier (Li et al., 2023). Oxidative stress levels are crucial for normal cell function, but hyperactivated oxidative stress may lead to further deterioration of intestinal barrier damage and epithelial cell death (Deng et al., 2025). Accumulating evidence has demonstrated that the pathogenesis of UC is critically associated with aberrant inflammatory responses, excessive oxidative stress, and gut microbiota dysbiosis, and these three factors are intricately interconnected (Tang et al., 2024). Specifically, gut microbiota dysbiosis can trigger intestinal inflammation and oxidative stress, which in turn further exacerbate microbial imbalance, forming a vicious cycle that drives UC progression (Wang Y. et al., 2023). The gut microbiome is a collective term for the various microorganisms, their genes, and their metabolites in the human gastrointestinal tract. In most healthy individuals, the gut microbiota consists mainly of the phylum Firmicutes and the phylum Bacteroidetes (Eckburg et al., 2005). The gut microbiota performs essential functions required for the maintenance of health, such as maintenance of gut barrier integrity (Shi et al., 2017), provision of nutrients (Nicholson et al., 2012), immunomodulation (Adak and Khan, 2019) and metabolism of pathogenic microorganisms (Wang J. et al., 2023). Gut microbiota dysbiosis occurs when the balance between the host and the gut microbiota is disturbed, such as when the diversity, composition and function of the gut microbiome are disrupted, which can adversely affect the host, thereby exacerbating immune imbalances and promoting the development of a variety of diseases, e.g., UC (Elinav et al., 2011). UC is a major focus of gut microbiota research. Gut microbes play an important role in maintaining the integrity of the intestinal mucosal barrier, defence against pathogen entry and leakage of pro-inflammatory metabolites (Wilson and Nicholson, 2017).

The American cockroach (*Periplaneta americana* L.), commonly known as the cockroach, is an insect of the genus Periplaneta in the family Blattidae, and is often used as a medicine in the form of dried carcasses or fresh insects, which were first recorded in China’s Shennong Ben Cao Jing (Classic of the Materia Medica of the Divine Husbandman) more than 2,000 years ago. Modern medical research has found that the cockroach has the ability to promote tissue repair, antioxidant, anti-inflammatory and enhance immune function (Li et al., 2015; Li et al., 2018). Kangfuxin (KFX) liquid is the extract of the dried insect body of American cockroach (Zhou et al., 2025), which is often used in the clinical treatment of UC by combining medication with retention enema, such as mesalazine combined with Kangfuxin liquid and Liuzasulfapyridine combined with Kangfuxin liquid. Currently, the mechanism of action of Kangfuxin liquid in the treatment of UC is not clear. The aim of this study was to investigate the effects of KFX on the level of systemic inflammation, intestinal barrier integrity, intestinal microbiota structure, and metabolic profiles of colonic contents in mice with DSS-induced ulcerative colitis using 16 S rRNA and non-targeted metabolomics to further explore the potential therapeutic mechanisms of KFX.

## 2 Materials and methods

### 2.1 Chemicals and reagents

Kangfuxin liquid was purchased from Inner Mongolia Jingxin Pharmaceutical Co. Ltd. (Inner Mongolia Autonomous Region, China). Dextran sodium sulfate (DSS, 35-50 kDa) was purchased from Meilun Bio (Dalian, China). Mesalazine enema was purchased from Dr. Falk pharma Gmbh (Germany).

### 2.2 Animal experiments

Forty C57BL/6 mice, male and female (age: 6–8 weeks, body weight: 20 ± 2 g) were purchased from the Experimental Animal Centre of Inner Mongolia Medical University (Inner Mongolia Autonomous Region, China). They were housed for 7 days under the following conditions: 12 h of light per day, temperature maintained at 24 °C–26 °C, relative humidity of 50%-70%, and provided with *ad libitum* water and food. All animal experimental procedures were approved by the Medical Ethics Committee of Inner Mongolia Medical University (No. YKD202503020).

At the end of acclimatisation feeding, mice were randomly divided into four groups: control group (C), model group (M), mesalazine group (MES) and KFX treatment group (KFX), with five females and males in each group. Animals in the control group were given *ad libitum* water. The other groups established the ulcerative colitis (UC) model by drinking water containing 2.5% DSS *ad libitum* for 7 days. DSS was dissolved in sterile drinking water at a concentration of 2.5% (w/v). The solution was replaced every 2 days and provided *ad libitum* for 7 consecutive days. In this study, all drug administrations were performed via enema. Animals in the MES group were treated with mesalazine enema solution (daily dose of 0.09 g/0.02 kg) daily for two consecutive weeks. Animals in the KFX group were treated with KFX liquid (daily dose of 100 mg/kg) daily for two consecutive weeks. Meanwhile, mice in groups Control and Model were treated with sterile saline given through the same mode of administration as in the MES and KFX groups.

To minimize sex bias, each group consisted of five female and five male mice. Before data analysis, we examined the interaction between sex and treatment using two-way ANOVA. No significant main effect of sex or sex-by-treatment interaction was observed for the primary outcome measures (DAI score, inflammatory cytokines, microbiota diversity; P > 0.05). Therefore, data from both sexes were combined for analysis.

### 2.3 Disease activity index (DAI)

During the experimental period, the activity and faeces of the mice were observed. In addition, the amount of diet and water consumed in each group and the weight of each mouse were recorded daily. Based on the amount of weight loss, faecal status and intestinal bleeding, a Disease Activity Index (DAI) score was derived and used to assess the severity of UC. The scoring criteria were as follows: weight loss: 0 (<2%), 1 (2%–5%), 2 (5%–10%), 3 (10%–15%), and 4 (>15%); faecal status: 0 (normal), 1 (soft/mucoid faeces), 2 (moderate diarrhoea/unshapely faeces), and 3 (diarrhoea/watery faeces); and haemorrhage: 0 (no blood), 1 (small amount of haemorrhage), 2 (markedly haematochezia), and 3 (heavy bleeding) (Wang et al., 2017).

### 2.4 Sample collection and preparation

Mouse serum was collected in sterile EP tubes for subsequent measurements of inflammatory cytokine levels and oxidative stress levels. Distal colonic tissue were collected in sterile EP tubes, one part was fixed and preserved in a solution of 4% paraformaldehyde for tissue pathology analysis and the other part was preserved in a −80 °C refrigerator for RT-PCR and Western blot analysis. Colon content samples were collected in sterile EP tubes and stored in a −80 °C refrigerator for 16S rRNA gene sequencing and metabolomics analysis.

### 2.5 Hematoxylin-eosin staining

To assess morphological changes in mouse colon, colon samples were embedded in paraffin and cut into thin slices, and then the sections were stained with hematoxylin and eosin (H&E) to assess histomorphology. Finally pathological changes in the colon tissue were assessed under light microscope and scored histologically. The scoring criteria are shown in Table 1 (Sann et al., 2013).

**TABLE 1:** Histological grading of colitis.

Score	Extent of inflammation	Crypt damage	Inflammation depth	Inflammatory infiltration area	Depth of lesion (non-inflammatory)
0	None	None	None	0	None
1	Slight inflammation	Low crypt deformation	Mucosal layer	0 ∼ 10%	Mucosal layer
2	Minor ulcer	Medium crypt deformation	Submucous laye	11% ∼ 25%	Submucous laye
3	Obvious ulcer	High crypt deformation	Muscular layer	26% ∼ 50%	Muscular layer and serosa layer
4	—	Entire crypt and epithelium lost	—	51% ∼ 100%	—

### 2.6 Enzyme-linked immunosorbent assay (ELISA)

The levels of inflammatory cytokines (TNF-α, IL-1β, IL-10) and oxidative stress markers (SOD, GSH-px, MDA) in mouse serum were quantitatively measured using ELISA kits, and the procedures were performed according to the manufacturer’s instructions (Nanjing Jiancheng Bioengineering Institute, Nanjing, China).

### 2.7 Real-time polymerase chain reaction (RT-PCR)

Total ribonucleic acid (RNA) was extracted from mouse colon tissue using TRIzol reagent (Thermo Fisher Scientific, Inc.). Afterwards, the extracted RNA was transcribed into total cDNA using the PrimeScript RT kit (Takara Bio, Inc.) and qPCR was performed using SYBR Premix Ex Taq II (Takara Bio, Inc.) according to the reagent vendor’s instructions. qPCR was performed using the SLAN automated PCR analysis system (8.2.2; Shanghai hongshi Medical Technology Co., Ltd.). Shanghai hongshi Medical Technology Co., Ltd.) to normalise the expression level of the target gene to that of β-actin. The primer sequences used in this study are shown in Table 2.

**TABLE 2:** Primer sequences for RT-PCR.

Primer name	​	Primer sequence (5′ to 3′)
β-actin	F	AGG​AGT​ACG​ATG​AGT​CCG​GC
	R	GGT​GTA​AAA​CGC​AGC​TCA​GTA
ZO-1	F	GAT​GTT​TAT​GCG​GAC​GGT​GG
	R	CAT​TGC​TGT​GCT​CTT​AGC​GG
Occludin	F	CAC​ACC​TCG​TCG​CTA​GTG​C
	R	AGA​TAA​GCG​AAC​CTG​CCG​A
Claudin-1	F	TGA​AGT​GCA​TGA​GGT​GCC​TG
	R	CAC​TAA​TGT​CGC​CAG​ACC​TGA​AA

### 2.8 Western blot analysis (western blot)

Mouse colon tissue proteins were extracted using RIPA lysate, protease inhibitor, and phosphatase inhibitor. Extracted proteins were separated by electrophoresis on a 10% sodium dodecyl sulfate-polyacrylamide (SDS-PAGE) gel and subsequently transferred onto a 0.45 μm pore size polyvinylidene difluoride membrane (Millipore, MA, United States of America). The membrane was closed with 5% skimmed milk powder for 1 h at room temperature and then incubated with primary antibody at 4 °C overnight. After several washes, the membrane was incubated with secondary antibody for 1 h at room temperature. Finally, protein bands were visualised using a luminescence imaging system (Tanon). The following primary antibodies were used: anti-ZO-1 (Abcam, Cambridge, CB, UK), anti-Occludin (Abcam, Cambridge, CB, UK), anti-Claudin-1 (Abcam, Cambridge, CB, UK) and anti-β-actin (Abcam, Cambridge, CB, UK) at a dilution of 1:1,000. The following secondary antibodies were used: goat anti-mouse IgG HRP (Abcam, Cambridge, CB, UK) and goat anti-rabbit IgG HRP (Abcam, Cambridge, CB, UK) at a dilution of 1:5,000.

### 2.9 Metabolomics of colon contents

Colon contents samples (50 mg) were weighed and 500 μL of pre-cooled methanol (containing 5 ppm 2-chlorophenylalanine) was added. The samples were mixed, vortexed and processed using ultrasound, followed by centrifugation at 12,000 rpm at 4 °C for 10 min, followed by taking the supernatant and passing it through a 0.22 μm filter membrane, and the filtrate was added to the assay vial. The sample filtrates were taken 10–20 μL each and mixed into one QC sample for evaluating the instrument stability and data reliability.

In this experiment, a high-resolution LC-MS platform from Thermo was used for non-targeted metabolomics of colon contents samples, and Compound DiscovererTM3.3 (version 3.3.3.31, Thermo, Waltham, United States of America) software was used in conjunction with a library of multiple types of MSMS profiles for metabolite qualitative and quantitative analyses. Compounds were separated on an ACQUITY UPLC HSS T3 column (100Å,1.8 μm, 2.1 mm × 100 mm) at a flow rate of 0.4 mL/min, with a column temperature of 40 °C, an autosampler at 8 °C, and a 2 μL injection volume.

DDA mass spectrometry data acquisition in positive and negative ion modes was performed using a Thermo Orbitrap Exploris 120 mass spectrometer under the control of the software Xcalibur (version: 4.7, Thermo), respectively. The offline raw format data were imported into the commercial software Compound DiscovererTM3.3 (version 3.3.2.31, Thermo, Waltham, United States). Metabolite identification was based on self-constructed libraries, mzCloud online library (https://www.mzcloud.org/), LIPID MAPS (https://www.lipidmaps.org/), HMDB (https://hmdb.ca/), MoNA (https://mona.fiehnlab.ucdavis.edu/), and the NIST_2020_MSMS mapping library were performed.

Expression abundance density plots were performed on metabolite abundance values using ggplot2 (V3.4.1). Principal Component Analysis (PCA), Partial Least Squares Discriminant Analysis (PLS-DA) were performed on the sample data using the R package Ropls, respectively. R2X and R2Y denote the explanatory rate of the constructed model for the X and Y matrices, respectively, and Q2 labels the predictive power of the model, and the closer their values are to 1 the better the model’s goodness-of-fit. The more the samples in the training set can be accurately classified into their original belongings. Metabolites with p-value <0.05 and VIP >1.5 in the PLS-DA model were considered as potential biomarkers. Impact-value >0.05 was used as a screening criterion for potential pathways. Differential substance enrichment analysis statistics are Bonferroni calibration results.

### 2.10 Sequencing of microbial diversity

Five randomly selected mice from each group were analysed for colon contents samples and genomic DNA of their gut microbes were extracted to further study the microbial community. PCR amplification was performed using 338F (5-barcode + ACT​CCT​ACG​GGG​AGG​CA-3) and 806R (5-GGACTACHVGGGTWTCTAAT-3) as primers in the V3 and V4 regions of the 16S rRNA gene. PCR amplification products were determined using 2% agarose gel electrophoresis, and PCR products were quantified on a Microplate reader (BioTek, FL × 800) using Quant-iT PicoGreen dsDNA Assay Kit and then on an Illumina NovaSeq platform for sequencing. Microbiome biology information was analysed using QIIME2 version 2019.4 with a modified and refined process according to the official tutorial (https://docs.qiime2.org/2019.4/tutorials/), which was performed as follows (Clua-Ferré et al., 2024) cutting primers using the cutadapt plugin; (Goens and Micic, 2020); using the DADA2 plugin for data processing such as quality filtering, denoising, splicing and chimera removal; The alpha diversity of the samples was described using the metrics of richness (Chao1), diversity (Shannon, Simpson), evenness (Pielou’s evenness) and coverage (Good’s coverage). The beta diversity of the samples was described using the PCoA metric. Microbial communities with significant differences between groups were detected by linear discriminant analysis of size (LEfSe). In addition, based on the KEGG database, microbial functions were predicted using PICRUSt2 (system genetic investigation of communities by reconstruction of unobobserved states) (Gavin M. Douglas, et al., preprint) predicted microbial function. ASVs were classified at the genus level. α/β diversity was calculated on the ASV-level characterisation table.

### 2.11 Statistical analyses

GraphPad Prism 9.5 (GraphPad Software, San Diego, CA, United States of America) software was used for statistical analysis. All experimental data were expressed as mean ± SD. Comparisons between two groups were performed using t-test. Comparisons among multiple groups were performed using one-way ANOVA followed by Tukey’s *post hoc* test. Statistical significance was considered when the P value was <0.05. The raw data supporting the conclusions of this article are available in the NCBI SRA repository [BioProject ID: PRJNA1423897].

## 3 Results

### 3.1 KFX ameliorated the symptoms of DSS-induced ulcerative colitis in mice

**FIGURE 1:**
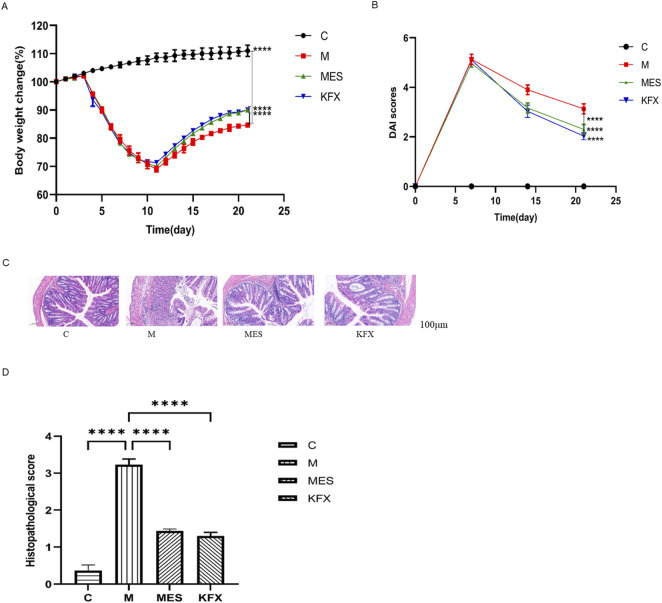
Effects of KFX on DSS-induced UC mice. **(A)** Body weight changes. **(B)** Disease activity index (DAI) score. **(C)** Representative image of HE staining of the colon tissues in each group (×100 magnification). **(D)** Histological scores of the colon calculated by H&E staining. Values are expressed as the means ± SD (n = 5). ^****^P < 0.0001 vs. Control group and ^****^P < 0.0001 vs. KFX group.

Mice in the experimental group exhibited symptoms such as diarrhoea, bloody stools, weight loss and, in severe cases, anal bleeding. In addition, mice in Model showed significant changes in body weight changes and DAI score (Figures 1A,B). Meanwhile, histopathology revealed pathological changes in the colonic tissues (Figure 1C), including severe damage to the colonic surface epithelium, destruction of crypt glands, reduction of goblet cells, and infiltration of a large number of inflammatory cells, which showed more severe colonic inflammation compared with the MES and KFX groups. Meanwhile, the histological score (Figure 1D) was significantly lower in the KFX. Therefore, KFX was able to reduce ulcerative colitis symptoms and ameliorate colonic histopathological injury in DSS-induced UC mice.

### 3.2 KFX reduces inflammation levels in UC mice

**FIGURE 2:**
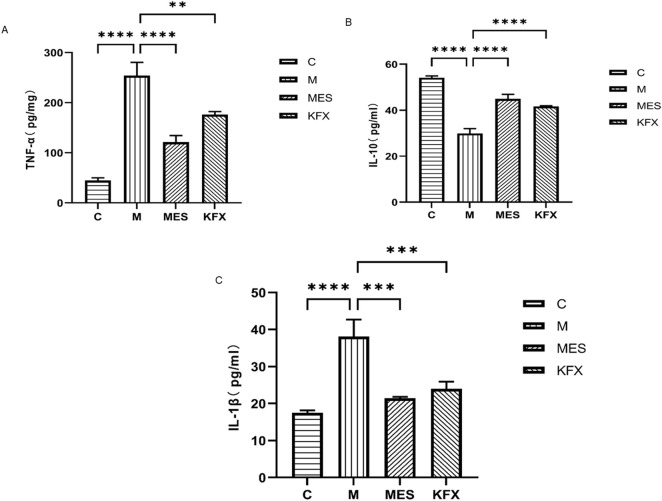
KFX reduced the inflammation level in UC mice. **(A–C)** Expression of TNF-α, IL-10 and IL-1β by ELISA. Values are expressed as the means ± SD (n = 5). ^**^P < 0.01 vs. KFX group, ^***^P < 0.001 vs. KFX group, ^****^P < 0.0001 vs. Control group and ^****^P < 0.0001 vs. KFX group.

To further investigate the effect of KFX on inflammation in UC mice, the pro-inflammatory cytokines tumour necrosis factor-α (TNF-α), interleukin-1β (IL-1β) and the anti-inflammatory cytokine interleukin-10 (IL-10) were detected (Mousa et al., 2025). The results showed that compared with Control, the expression levels of TNF-α and IL-1β were significantly higher in the serum of mice in M, while the level of IL-10 was lower. Compared with Model, the expression levels of TNF-α and IL-1β were significantly lower, while IL-10 levels were significantly higher in the serum of mice in the KFX (Figures 2A–C). The experimental results showed that KFX had a significant therapeutic effect on UC, and KFX improved the pathological state and inflammation level of DSS-induced UC mice.

### 3.3 KFX inhibits oxidative stress injury in UC mice

**FIGURE 3:**
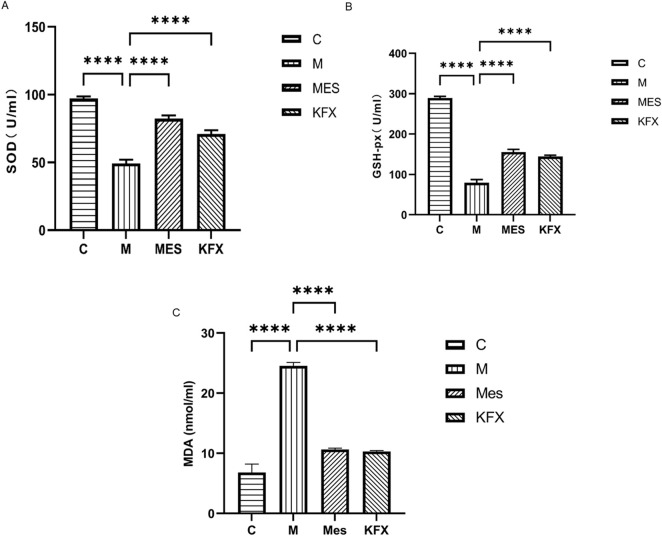
KFX inhibits oxidative stress injury in UC mice. **(A)** SOD activity; **(B)** GSH-px activity; **(C)** MDA levels. Values are expressed as mean ± SD (n = 5). **P < 0.01, ***P < 0.001, ****P < 0.0001.

Previous studies have shown that the onset and progression of many intestinal diseases are associated with reactive oxygen species and free radicals (Li and Wang, 2025). SOD and GSH-px are endogenous antioxidant enzymes in the body, which act to eliminate reactive oxygen species. Therefore, the ability of the body to resist oxidative stress can be reflected by the activity of these enzymes. The results showed that compared with the control group, the serum levels of SOD and GSH-px were significantly decreased, while the level of MDA was significantly increased in the model group. Compared with the model group, the expression levels of SOD and GSH-px were significantly higher, and the level of MDA was significantly lower in the KFX group (Figures 3A–C). The experimental results indicated that oxidative damage was aggravated in mice induced by DSS, and the intervention of KFX inhibited the aggravation of oxidative stress damage in UC mice.

### 3.4 KFX strengthens the intestinal barrier in UC mice

**FIGURE 4:**
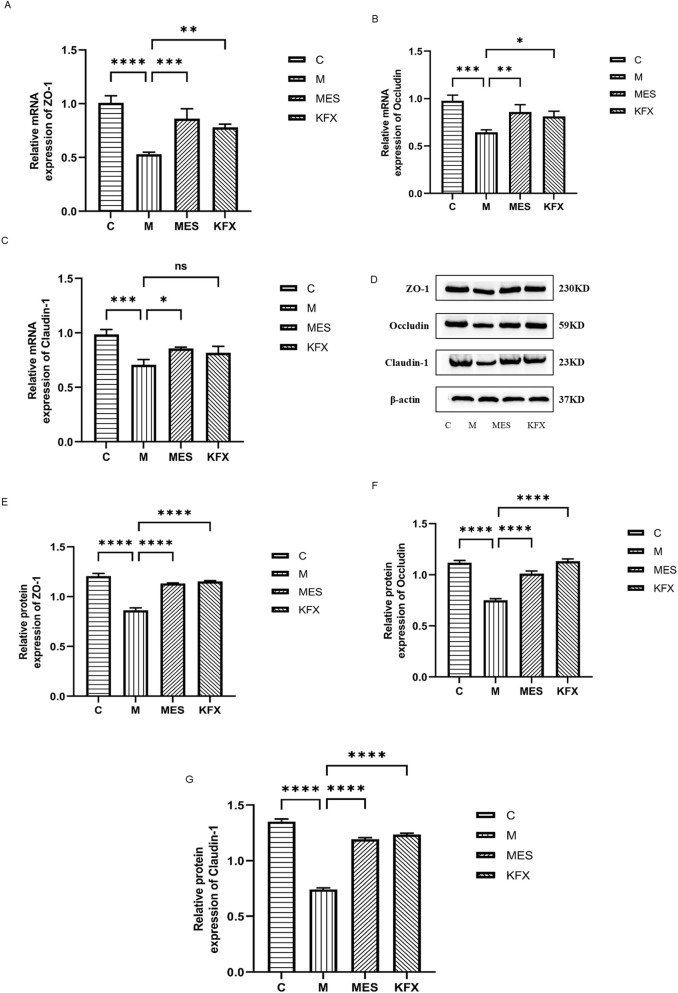
KFX strengthens the intestinal barrier in UC mice. **(A–C)** Expression of ZO-1, occludin and claudin-1 detected by RT-PCR. **(D–G)** Expression of ZO-1, occludin and claudin-1 detected by Western blot. Values are expressed as the means ± SD (n = 5). ^**^P < 0.01 vs. KFX group, ^***^P < 0.001 vs. KFX group, ^****^P < 0.0001 vs. Control group and ^****^P < 0.0001 vs. KFX group.

Intestinal barrier function is closely related to the occurrence and development of UC(29). To investigate the effect of KFX on intestinal barrier function, we examined the expression of intestinal barrier-related proteins using RT-PCR and Western blot. The tight junction proteins ZO-1, occludin and claudin-1 play key roles in maintaining the integrity and cell permeability of the intestinal epithelial barrier (La et al., 2025). The results showed that the expression levels of ZO-1, occludin and claudin-1 were significantly reduced in the colonic tissues of Model mice compared with Control mice. The expression levels of ZO-1, occludin and claudin-1 were significantly higher in the colonic tissues of mice in the KFX compared with mice in the Model (Figures 4A–G). The experimental results indicated that KFX upregulated the expression of tight junction proteins and enhanced intestinal barrier integrity at the molecular level in UC mice.

### 3.5 KFX affects metabolites and potential metabolic pathways in UC mice

**FIGURE 5:**
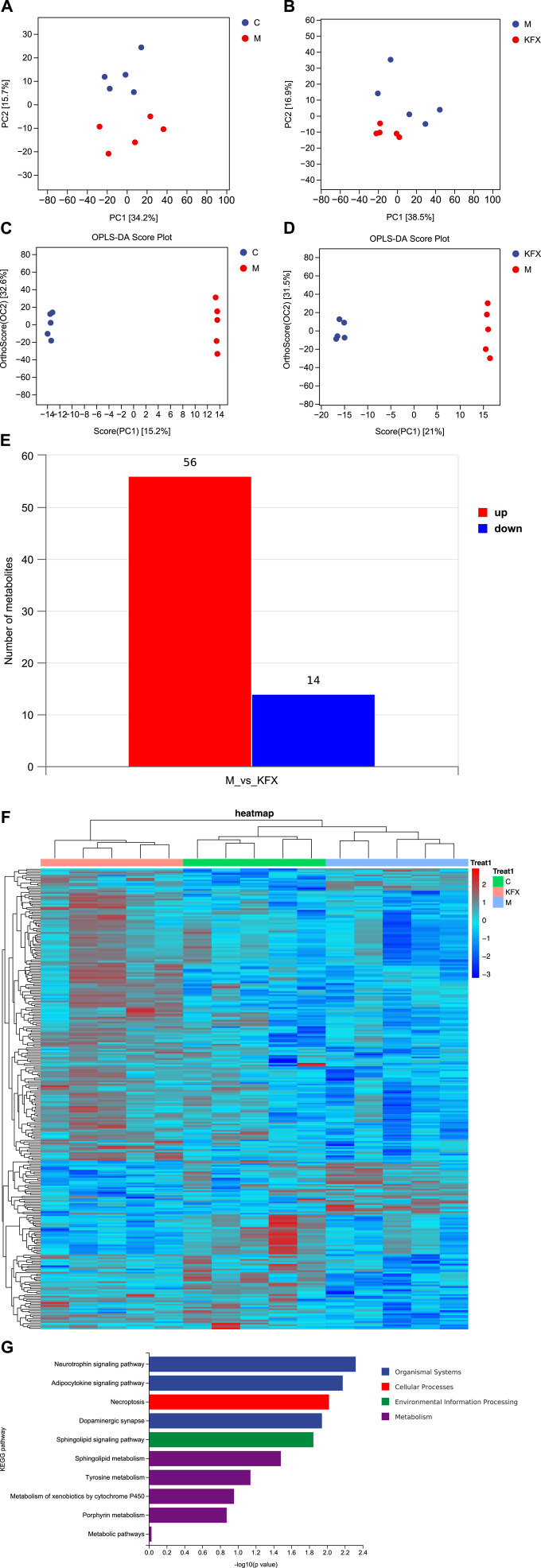
Results of untargeted metabolomics analysis. **(A,B)** PCA core plot of Control vs. Model, and Model vs. KFX group. **(C,D)** OPLS-DA score plot of Control vs. Model (R^2^Y = 1, Q^2^ = 0.693), and Model vs. KFX group (R^2^Y = 0.999, Q^2^ = 0.663). **(E)** Differential substance screen (Model vs. KFX). **(F)** Correlation analysis of differential substances. **(G)** Differential material enrichment analysis.

To explore alterations in metabolites and metabolic pathways, we examined the metabolite composition of colonic contents in the Control, Model and KFX by untargeted metabolomics analysis. Principal Component Analysis (PCA) and Partial Least Squares Discriminant Analysis (PLS-DA) were performed on the metabolites screened in the positive ion model, respectively. PCA and PLS-DA revealed differences between the groups. Data analyses (Figures 5A–D) showed a clear separation between the Control vs. Model group and the Model vs. KFX group, and the Model vs. MES group, suggesting that the model had been established. The cross-validation parameters (R2Y and Q2) between the Control and Model and between the Model and KFX were all greater than 0.6, which demonstrated that the OPLS-DA model and the quality of the data were reliable. Based on VIP values (VIP >1.5) and t-tests (P < 0.01), 70 potential metabolites were identified in UC mice treated with KFX (Figure 5E). The correlations of differential substances are shown as a heatmap (Figure 5F). These metabolites were mainly related to Lipid metabolism (Ceramide (d18:1/9Z-18:1)), Amino acid metabolism (Homovanillic acid), Xenobiotics biodegradation and metabolism (1- Nitronaphthalene-5,6-oxide) and Metabolism of cofactors and vitamins (urobilin) (Figure 5G). The analysis revealed that the mechanism of KFX treatment for UC involves specific metabolic pathways, such as sphingolipid metabolism, tyrosine metabolism, metabolism of xenobiotics by cytochrome P450 and porphyrin metabolism. Taken together, the experimental results suggest that alterations in metabolites and metabolic pathways may play a key role in the protective effect of KFX against UC.

### 3.6 KFX modulates gut microbiota dysbiosis in UC mice

**FIGURE 6:**
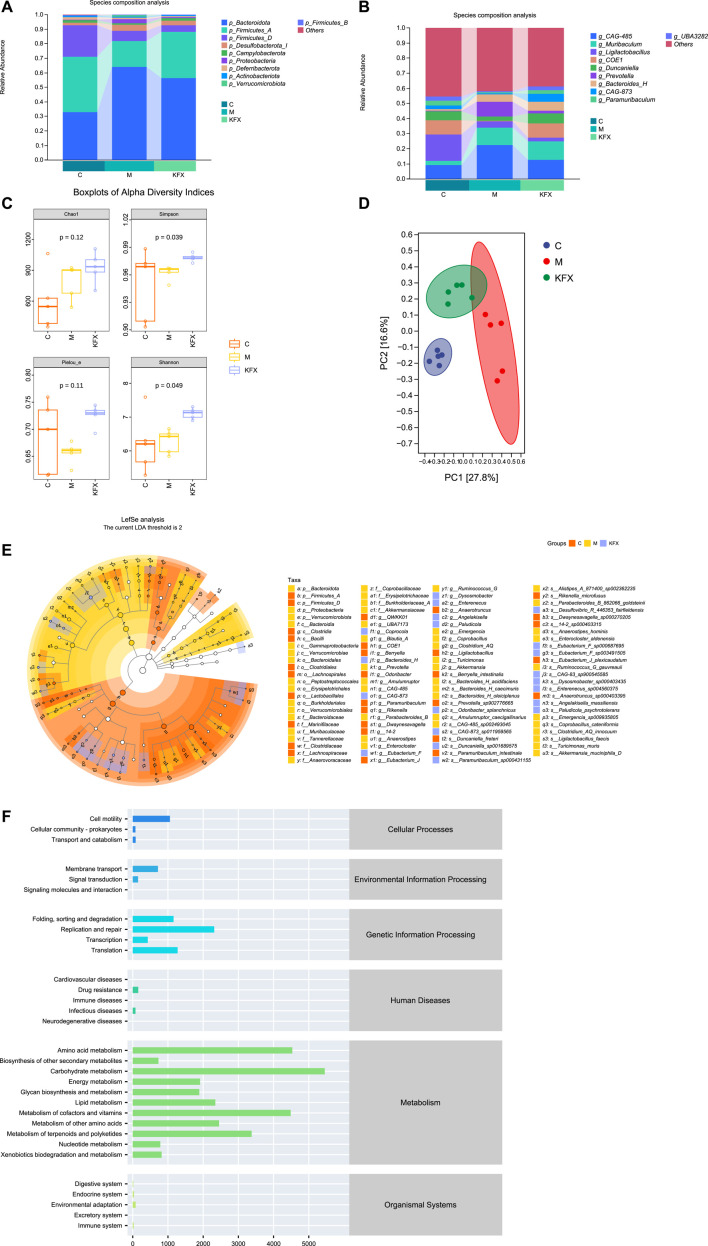
Results of 16S rRNA analysis. Relative abundance of gut microbiota at the phylum **(A)** and genus **(B)** levels in three groups revealed by 16S rRNA sequencing (different colors represent different bacteria at phylum or genus levels). **(C)** Alpha Diversity Index among Control, Model and KFX groups. **(D)** Principal coordinate analysis score (PcoA) analysis among Control, Model and KFX groups. **(E)** Differences in microbiota composition between three groups with Linear discriminate analysis Effect Size (LEFSe). **(F)** KEGG functional prediction analysis of differential microbe.

Species composition analyses (Figures 6A,B) revealed the top ten microorganisms with the highest relative abundance at the phylum and genus levels. At the phylum level, the predominant gut microbial components included the Bacteroidota and the Firmicutes. Compared to Control, the gut microbial composition in Model showed a decrease in the content of the Firmicutes and an increase in the content of the Proteobacteria. However, the compositional trend was reversed after KFX treatment. Ten genera were identified in the intestinal flora of mice, with significant changes in the relative abundance of *Prevotella*, *CAG-485*, *paramuribaculum* and *COE-1* genera in the three groups. The richness index (Chao), diversity index (Shannon and Simpson) and evenness index (Pielou) (Jiang et al., 2025) were used for α-diversity analysis (Figure 6C). Simpson and Pielou indices were lower in Model compared to Control. Chao, Shannon, Simpson, and Pielou indices were higher in KFX compared to Model. Principal coordinate analysis (PCoA) was used for the beta diversity analysis (Figure 6D), and the results showed a clear separation between Control, Model and KFX. Analyses based on linear discriminant analysis of effect size (LEFSe) were used and visualised in the form of taxonomic branching diagrams to detect microbiota of different abundance between these groups (Figure 6E). Each individual circle represents each level of microbiota, and small circles of different diameters represent gut microbes with different relative abundances. Higher LDA scores indicate significant differences in that microbiota. The top 98 gut bacteria occur at different levels of the LDA score> 3.0. Notably, 33 microbial genera that differed significantly between groups were identified and were in high abundance in the sample. Predictive analyses of microbial function showed that the main changes occurred in carbohydrate metabolism, amino acid metabolism, and the metabolism of cofactors and vitamins (Figure 6F). In summary, the experimental results suggest that the regulation of gut microbiota may play a key role in the protective effect of KFX against UC.

### 3.7 Correlation between metabolites and microbiota composition

**FIGURE 7:**
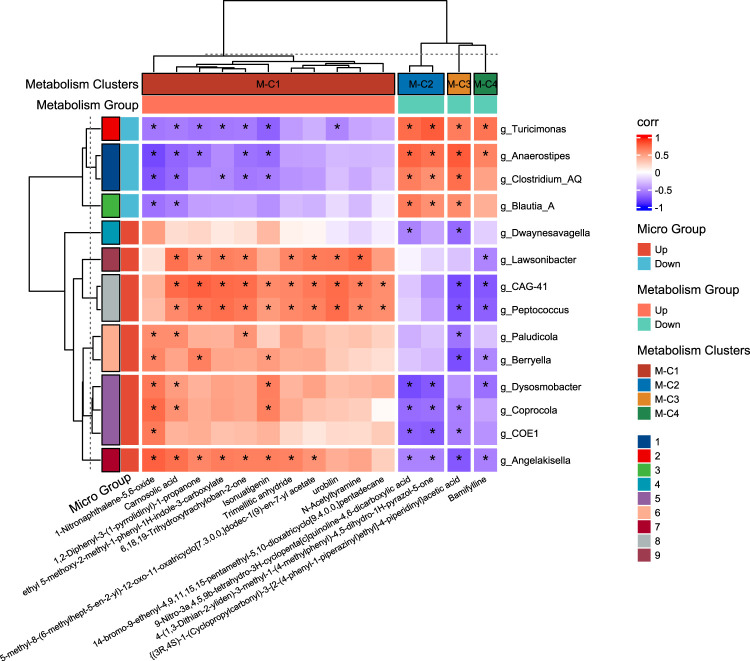
Heat map of the correlation between metabolites and microbiota composition in DSS-induced ulcerative colitis mice (^*^P < 0.05).

Spearman’s rank correlation analysis was performed to determine the potential associations between microbial genera in the colonic contents of DSS-induced ulcerative colitis mice and metabolites. As shown in Figure 7, among the Up-group genera, *CAG-41* was significantly positively correlated with urobilin and carnosolic acid. *Peptococcus* showed a significant positive correlation with urobilin and isonuatigenin. *Lawsonibacter* was significantly positively correlated with N-Acetyltyramine and carnosolic acid. Among the Down-group genera, *Anaerostipes* was significantly negatively correlated with carnosolic acid. *Turicimonas* showed significant negative correlations with urobilin and isonuatigenin. *Clostridium-AQ* was significantly negatively correlated with carnosolic acid.

## 4 Discussion

Ulcerative colitis is a chronic, non-specific, recurrent inflammatory gastrointestinal disease, with an increasing incidence worldwide, imposing a heavy burden on patients’ lives and healthcare systems (Ungaro et al., 2017). In this study, using a DSS-induced UC mouse model, we systematically investigated the therapeutic effects and underlying mechanisms of *P. americana* extract on UC, providing a solid scientific basis for the clinical application of KFX. Our results showed that KFX enema treatment significantly alleviated DSS-induced body weight loss, elevated DAI scores, and colon shortening in UC mice. HE staining revealed that KFX markedly improved colonic histopathological damage and reduced inflammatory cell infiltration in UC mice. This finding is consistent with reports on the protective effect of *P. americana* extract against acetic acid-induced UC in rats, confirming the good therapeutic potential of KFX for UC (Li et al., 2016).

Pro-inflammatory cytokines play a key role in the pathogenesis and progression of UC (Shi et al., 2025). These signalling molecules produced by various cell types orchestrate the inflammatory response that leads to mucosal damage. TNF-α and IL-1β are two pro-inflammatory cytokines in UC that can lead to a worsening of the inflammatory response. Therefore, targeting them with specific inhibitors or antibodies has been shown to improve UC symptoms (Straatmijer et al., 2023). For example, anti-tumour necrosis factor-α (TNF-α) inhibitors and antibodies have been shown to be effective in reducing inflammation in patients with UC. In addition, IL-1β inhibitors have not been used in the treatment of UC. In this study, we examined the levels of pro-inflammatory cytokines TNF-α and IL-1β, which are highly associated with UC. The levels of TNF-α and IL-1β were significantly reduced in mice in the KFX group compared with the Model group. This suggests that KFX can attenuate the inflammatory response in UC.

Reactive oxygen species (ROS), as components affecting intestinal homeostasis, are signalling molecules widely involved in numerous cellular processes (Bhattacharyya et al., 2014). Pathologically, mitochondria produce and accumulate reactive oxygen species (ROS). At the same time, the redox system in the body keeps the cells in redox balance. However, the inflammatory response usually promotes the production of ROS, which in turn amplifies the inflammatory response and triggers the release of inflammatory mediators such as IL-1β, TNF-α, and IFN-γ, thus triggering a vicious cycle (Forrester et al., 2018). Oxidative stress, in combination with multiple factors including genetic susceptibility, alterations in the intestinal epithelium, dysregulated immune response and microbiota intolerance, affects the onset and progression of UC (Tian et al., 2017). MPO activity is associated with oxidative damage and severity of UC (Schroder et al., 2022), and previous studies have shown that MDA reflects the levels of lipid peroxidation and oxidative inflammatory factors, and the higher the MDA level, the more severe the oxidative damage in colonic tissues (Li et al., 2025). The results of the present experiment showed reduced SOD activities, revealing impaired function of antioxidant enzyme systems in the UC model, and decreased levels of GSH-px, which suggests a reduction in endogenous antioxidant reserves. Furthermore, the significantly reduced serum MDA levels in the KFX group further confirm that KFX alleviates DSS-induced lipid peroxidation injury. The above findings suggest that KFX can significantly inhibit oxidative damage and inflammatory factor accumulation by restoring endogenous antioxidant functions.

Intestinal barrier dysfunction is an important pathological feature of UC. The tight junction proteins claudin-1, occludin, and ZO-1 are key molecules that maintain the intestinal mechanical barrier, and their downregulated expression leads to increased intestinal permeability (Yoon et al., 2017). This study found that KFX significantly upregulated the mRNA and protein expression levels of these tight junction proteins in the colonic tissue of UC mice. These findings suggest that KFX can repair the intestinal mechanical barrier and alleviate UC symptoms.

A healthy gut environment is maintained by regulating the balance of the gut microbiota, metabolites and the host immune system. However, changes in the microbiota can disrupt the beneficial interactions between the microbiota and the host, which can lead to disease (Tan et al., 2025). Changes in the host’s metabolism can both affect and be affected by the state of the gut microbiota, which in turn affects the severity of inflammation and the state of the colonic mucosa (Yuan et al., 2021). The intestinal microbiota plays a crucial role in modulating the colonic immune response and enhancing the intestinal barrier function through the production of short-chain fatty acids, bile acids, and a variety of bioactive molecules (Zhang et al., 2025). The 16 S rRNA results showed that after KFX treatment, the relative abundance of two potentially harmful bacteria (*Prevotella* and *CAG-485*) decreased. Elevated *Prevotella* abundance has been linked to increased intestinal permeability and pro-inflammatory cytokine production in DSS-induced colitis (Fan et al., 2024), while *CAG-485* is a mucin-degrading bacterium whose overgrowth may compromise the mucus barrier (Hao et al., 2025). While the relative abundance of two potentially beneficial bacteria (*Paramuribaculum* and *COE-1*) increased. Of note, *Paramuribaculum* has been reported to be positively associated with short-chain fatty acid production in murine colitis models (Feng et al., 2022), suggesting a potential protective role in intestinal homeostasis. Currently, this is still a new nine that has not yet been formally named or published, and is commonly referred to as “Lachnospiraceae bacterium COE1” (Wang et al., 2018). The Lachnospiraceae family is a very important group of commensal bacteria in the human gut, known for producing short-chain fatty acids (such as butyrate) (Wang et al., 2024). These results suggest that KFX restored the balance of intestinal microorganisms by modulating intestinal flora and significantly alleviated DSS-induced colitis.

The metabolomics results suggest that the mechanism of KFX treatment of UC involves specific metabolic pathways such as sphingolipid metabolism, tyrosine metabolism, cytochrome P450 metabolism to xenobiotics and porphyrin metabolism. In the sphingolipid metabolism pathway, ceramide is a pro-apoptotic sphingolipid signaling molecule (Kitatani et al., 2008). Previous studies have shown that elevated ceramide levels in the colonic mucosa of UC patients can induce intestinal epithelial cell apoptosis by activating the mitochondrial apoptotic pathway (e.g., increased Bax/Bcl-2 ratio and caspase-3 activation), thereby disrupting tight junction proteins (ZO-1, Occludin) and increasing intestinal permeability (Leucht et al., 2014). In our study, ceramide was significantly reduced after KFX treatment, accompanied by upregulated ZO-1 and Occludin expression (Figure 4), suggesting that KFX may repair the intestinal barrier by inhibiting ceramide-mediated apoptosis. Furthermore, homovanillic acid in tyrosine metabolism, an oxidative stress marker (Sodhi et al., 2021), was decreased in the KFX group, consistent with restored SOD and GSH-px activities (Figure 3).

Up-group genera such as *CAG-41*, *Peptococcus*, and *Lawsonibacter* showed significant positive correlations with multiple metabolites that possess known biological activities. Urobilin (尿胆素) is the terminal product of heme degradation, generated by the bacterial reduction of bilirubin in the gut (Williams et al., 2025). The positive correlation of *CAG41* and *Peptococcus* with urobilin suggests that these bacteria may participate in bilirubin metabolism, which is closely linked to inflammation and oxidative stress (Bates et al., 2023). N-Acetyltyramine is a tyramine derivative that can be produced by microbial decarboxylation of tyrosine followed by acetylation (Ran et al., 2022). The positive correlations of *CAG-41* and *Lawsonibacter* with N-Acetyltyramine indicate that these genera may be involved in aromatic amino acid metabolism. Carnosolic acid is a diterpene compound with anti-inflammatory and antioxidant properties (Chen et al., 2024). The positive correlations of *CAG-41*, *Peptococcus*, and *Lawsonibacter* with carnosolic acid suggest that these bacteria may promote its accumulation or, conversely, be enriched by it. Down-group genera, including *Anaerostipes*, *Turicimonas*, and *Clostridium-AQ*, were negatively correlated with the above-mentioned metabolites. *Anaerostipes* is a known butyrate-producing genus; its decrease after KFX treatment and its negative correlations with multiple metabolites may reflect alterations in the gut microenvironment (Liu et al., 2024). Previous studies have shown that *Turicimonas* is associated with inflammation (Cao et al., 2025). Its negative correlations with urobilin and isonuatigenin suggest that these metabolites may inhibit its growth, or that its reduction leads to decreased accumulation of these metabolites. *Clostridium-AQ* is often altered in UC (Geng et al., 2025), and its negative correlation with carnosolic acid implies a potential antagonistic relationship between this bacterium and protective metabolites. Therefore, KFX may reshape the gut microbiota by enriching beneficial bacteria that are positively associated with anti-inflammatory and antioxidant metabolites, while suppressing bacteria that are negatively correlated with these metabolites.

Our experiments showed that KFX exhibited comparable or even unique effects to MES in relieving colitis, suggesting partially overlapping but complementary mechanisms. As a multi-component natural extract (Ma et al., 2022), KFX possesses additional advantages likely stemming from its multi-targeting properties. For instance, KFX simultaneously coordinates the microbe-metabolite-host barrier axis.

Overall, KFX may restore the damaged intestinal barrier function by modulating the dysregulated flora (reducing harmful bacteria and increasing beneficial bacteria) towards a healthy state, which in turn reverses the DSS-induced disruption of multiple metabolic pathways, such as sphingolipid metabolism, and KFX may inhibit excessive apoptosis of intestinal epithelial cells by affecting the balance of signalling molecules, such as ceramides, thus restoring the damaged intestinal barrier function. KFX may through synergistic multiple metabolic pathway modulation to achieve the combined effects of inhibiting inflammation, alleviating oxidative stress, and repairing the intestinal epithelial barrier, thereby significantly alleviating ulcerative colitis. However, more studies are needed to obtain more comprehensive and reliable conclusions. Therefore, we will further analyse the potential mechanism of KFX for UC using transcriptomics in the future.

This study has several limitations. First, although we observed KFX-induced modulation of gut microbiota and metabolites, the causal role of the microbiota has not been validated by fecal microbiota transplantation or germ-free animal models. Second, multi-omics correlation analyses were limited to descriptive statistics, without integrated network analysis or mediation effect tests. Third, only a single dose (100 mg/kg) and a single administration route (enema) were evaluated; the optimal dose and regimen need further exploration. Fourth, the effects of KFX were assessed only after 2 weeks of treatment; long-term safety and efficacy remain to be investigated. Fifth, inflammatory cytokines were measured only in serum rather than in colonic tissue. We acknowledge that measuring cytokines in colonic tissue would provide complementary information regarding local inflammatory responses. Therefore, future studies should include both serum and colonic tissue cytokine analysis to more comprehensively understand the mechanism of action of KFX. Furthermore, although no significant sex differences were detected, the sample size per sex (n = 5) may be underpowered to detect smaller sex-specific effects. Future studies should use larger sample sizes or specifically designed sex-stratified analyses.

## 5 Conclusion

By integrating colon content metabolomics and 16S rRNA gene sequencing, this study reveals the ameliorative effects of KFX on DSS-induced UC in mice and its association with the gut microbiota-metabolite network. KFX intervention significantly reduced body weight loss and disease activity index (DAI), suppressed colonic inflammation and oxidative stress, and repaired the intestinal mucosal barrier (evidenced by upregulated ZO-1 and Occludin). KFX decreased the abundance of potentially harmful bacteria genera associated with colitis (e.g., *Prevotella*, *CAG-485*) and increased the abundance of commensal bacteria genera linked to short-chain fatty acid production (e.g., *Paramuribaculum*, *COE-1*). KFX reversed UC-related metabolic disturbances, particularly affecting sphingolipid metabolism (reducing pro-apoptotic ceramide) and tyrosine metabolism (reducing the oxidative stress marker homovanillic acid), which are consistent with intestinal barrier repair and redox balance restoration. In conclusion, KFX exerts significant protective effects and these effects are accompanied by favorable alterations in gut microbiota composition and metabolic profiles. These findings suggest that the gut microbiota-metabolite axis may represent a potential pathway contributing to the therapeutic action of KFX, suggesting its potential as a candidate drug for the clinical treatment of UC.

## Data Availability

The raw data supporting the conclusions of this article are available in the NCBI SRA repository (BioProject ID: PRJNA1423897).
